# Healing of the Achilles tendon in rabbits—evaluation by magnetic resonance imaging and histopathology

**DOI:** 10.1186/s13018-014-0132-1

**Published:** 2014-12-12

**Authors:** Wilson Campos Tavares, Ubiratam Brum de Castro, Eduardo Paulino Jr, Leonardo de Souza Vasconcellos, Ana Paula Madureira, Maria Angélica Baron Magalhães, Daniel Victor Moreira Mendes, Adriana Maria Kakehasi, Vivian Resende

**Affiliations:** Musculoskeletal Section of Clinics Hospital of Universidade Federal of Minas Gerais (UFMG), Rua Goncalves Dias, 750 Apto, 1803 Funcionarios, CEP 30140091 Belo Horizonte, Minas Gerais Brazil; Radioclinica Itauna, Itauna, Minas Gerais Brazil; Faculty of Medicine, UFMG, Belo Horizonte, Minas Gerais Brazil; Faculty of Medicine, UFSJ, São João del-Rei, Minas Gerais Brazil

**Keywords:** MRI, Tendon injury, Experimental study, Perfusion MRI

## Abstract

**Background:**

Dynamic contrast-enhanced magnetic resonance imaging (DCE-MRI) could provide valuable findings for tendon regeneration. A non-invasive image method that can effectively evaluate the quality of the scar tissue has not yet been employed.

**Methods:**

Thirteen New Zealand rabbits were divided into two groups: group 1—non-treated control (*n* = 4); group 2—surgical intervention (*n* = 9). The central portion of the Achilles tendon was resected, and after 30 days, DCE-MRI was performed. Contrast enhancement methods were applied using the region of interest (ROI) technique. In the medium third of the Achilles tendon, the intra-substantial signal intensity and the presence of hyper-intense intra-tendon focus points and of signal heterogeneity were evaluated. Antero-posterior and transversal diameters of the tendon were measured. The Achilles tendon was removed and dissected free from other tissues. Sections from the central part of the tendon were stained for histological analysis.

**Results:**

The difference between the contrast enhancement curves of the control and surgical groups (*p* < 0.0001) was observed. The surgical group had an intense contrast enhancement in the contrast sequences, enlargement of the diameter and intra-substantial signal intensity alteration, with hyper-signal focus points and widening of the tendon sheath, which presented irregular contours and intense contrast enhancement. On histology, the Achilles tendon presented diffuse widening of the tendon sheath and wedge-shaped areas with scarring tissue rich in disordered collagen fibres. These findings were related to alteration in the intra-substantial signal intensity, with hyper-signal focus points in the DCE-MRI.

**Conclusions:**

MRI with perfusion could be a useful technique for evaluating tissue and fibrous scarring in tendons.

## Introduction

Rupture of the Achilles tendon has increased, which is probably associated with the practice of high-demand sports that put a great load on it. Most commonly, this occurs with men above the age of 40 years. The best course of treatment remains controversial and full recovery may take over a year. Many patients never regain full strength and function, even with intense rehabilitation [1].

As demonstrated during the last years, rehabilitation accelerated by early deambulation is important for the post-operative recovery of the Achilles tendon [2,3]. The success of this therapy is based on its capacity to minimize the risk of new rupture and other complications [4]. A non-invasive image method that can effectively evaluate the quality of the scar tissue has not yet been employed.

There is an increasing interest in the use of magnetic resonance imaging (MRI) to control and follow the healing of the Achilles tendons [5]. Dynamic contrast-enhanced magnetic resonance imaging (DCE-MRI) has been recently used as a method of investigation for many diseases, among which those related to the osteomuscular system, specially tumours [6] and arthropathies [7,8], but has very rarely been employed in the study of tendinosis [9].

DCE-MRI is a physiological imaging method to evaluate the enhancement of the contrast agent and entails the pharmacokinetic characterization as the contrast agent enters and exits the region under investigation (e.g. an individual voxel or a region of interest (ROI)). As sequences of MRI are done during and immediately after bolus injection of the contrast agent, they reveal the initial distribution of the contrast agent in the capillaries and the interstitial tissue and allow for the generation of a signal intensity time course from a tissue of interest which can then be analyzed qualitatively or quantitatively to provide characterization of various features of that tissue’s physiology [10]. This technique provides information regarding the tissue vascularization, capillary perfusion and permeability. Images obtained through DCE-MRI represent a technique of dynamic evaluation during the post-contrast phase, which may supply information about the tendon’s vascular permeability.

The aim of this paper was to evaluate the Achilles tendon in rabbits, employing DCE-MRI and histological techniques as a method of assessing the healing of the tendon.

## Method

Thirteen mature New Zealand white male rabbits were used. The rabbits were housed one per cage with food and water *ad libitum*. They were divided into two groups: group 1 (*n* = 4) = untreated control group and group 2 (*n* = 9) = surgical intervention group in the Achilles tendon. This study was approved by the Animal Experimentation Ethics Committee (CETEA) of the Federal University of Minas Gerais (protocol number 183/2012).

**Figure 1:**
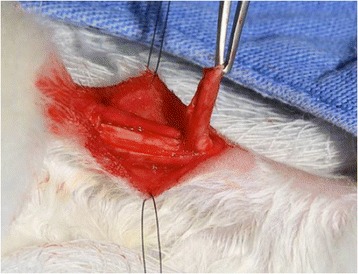
**Surgical procedure in the rabbit’s Achilles tendon: resection of the central segment of the tendon.**

Animals were anaesthetized with an intramuscular injection of 3.5 mg/kg of xylazine (Calminum, Agener União, Brasil) and 6 mg/kg of ketamine (Ketamina Agener 10%, Agener União, Brasil). The central third of the tendon was excised, which was 3 mm wide and 10 mm long (Figure 1) [11]. In order to identify the location of the resected portion, four markers were placed at the corners of the defect with 5-0 nylon. Next, the tendon sheath (paratendon) was closed with a continuous 6.0 prolene suture and the skin with a continuous 3.0 nylon suture. No immobilization was applied after surgery, and the rabbits were allowed unrestricted activities in their cages. They received dypirone at 25 mg/kg as post-operative analgesia.

MRI was initially performed in the control group and 4 weeks later in the surgical intervention group. For this procedure, the animals were anaesthetized under the same protocol previously described. Studies were carried out in an HDxt Signa GE 1.5 Tesla magnetic resonance machine (GE Healthcare, Milwaukee, WI, USA). In the right lateral decubitus position, the right posterior member of the rabbit was placed in a 9-channel knee coil (one transmitter and eight receivers). The protocol consisted of an axial and sagittal T2 sequence with fat suppression: TR/TE: 5,000/75 ms; 2.5-mm-wide cut; matrix 320 × 256; field of vision (FOV): 100 mm; number of excitations (Nex): 2; and of an axial T1-weighted sequence: TR/TE: 700/15 ms; 2-mm-wide cut; matrix 320 × 256; FOV: 120 mm; Nex: 2.

Dynamic contrast-enhanced perfusion used in the axial plan was the 3D spoiled gradient recalled sequence with a 1.2-mm-wide cut, TR/TE 2/7 ms, “*flip angle 8*”, one acquisition, FOV of 200 mm, 128 matrix and 20 s. These sequences were composed of 30 slices, after a bolus injection of a 0.3 mg/kg dose of gadopentetate dimeglumine (469 mg/ml, Magnevist, Schering, Berlin, Germany). A bolus contrast agent was infused at a speed of 1.5 ml/s, followed by a saline solution infusion (10 ml). Contrast injection was initiated immediately after the first sequence without contrast of the dynamic series. Five dynamic sequences, without intervals, were initiated together with the contrast injection. All the images of dynamic enhancement were obtained after the first spoiled gradient recalled sequence pre-contrast.

In the post-contrast images, a minimal limit for the exclusion of signals was established, in order to keep out of the final analyses of all the pixels outside the tendon region or pixels with a low signal that could interfere with the analysis. Furthermore, all the pixels that presented a poorly defined curve (with considerable signal intensity oscillations) were excluded, as well as all image devices that could affect the quality of the classification.

MR images were analyzed by a blind trained examiner with 6 years of experience in musculoskeletal MRI. In the phase without perfusion, the intra-substantial signal intensity and the presence of hyper-intense intra-tendon focus points and of signal heterogeneity in the medium third of the Achilles tendon were evaluated. In the T1- and T2-weighted sequence, antero-posterior and transversal diameters were measured.

In the phase with perfusion, the artery was firstly analyzed. Pixels with higher intensity difference were identified, and subsequently, the perfusion curve of the medium third of the tendon was evaluated. The intra-tendon area that could correspond to the healing process was identified. Contrast enhancement methods were applied using the region of interest (ROI) technique. ROI was placed at the same distance from the insertion of the Achilles tendon in all the MRI studies. Circular ROI, with a 2-mm diameter, was placed in the area with greater enhancement in the coloured map study, avoiding the vascular plexus and the tendon sheath. When no contrast enhancement was found, the measures were taken from the centre of the tendon.

Analyses were carried out with *workstation ADW 4.6 GE functool* by two experienced radiologists, who used the contrast to compare the enhancement curve patterns.

After the MRI, the animals were sacrificed with an overdose of 10% KCL solution under general anaesthesia. The entire Achilles tendon was removed and dissected free from other tissues. The specimens were fixed in a buffered 10% formalin solution for 24 h and cast in paraffin blocks. Sections from the central part of the tendon were stained with haematoxylin and eosin.

The slides were scanned with a Panoramic MIDI (3DHISTECH, Budapest, Hungary). Using the images obtained, the antero-posterior and transversal diameters of the tendon were measured. Slides were analyzed by an experienced examiner, with no knowledge of the group to which the animal belonged. The analysis consisted in identifying granulation tissue and fibroblast arrangements in the tendon tissue, capillaries and inflammatory cells. In order to evaluate if there was any correspondence between the histological and the MRI findings, the transversal sections of both were placed side by side.

“D’Agostino & Pearson omnibus normality test” was used to verify the normality of the sample errors and to test the asymmetry and kurtosis values. Variables with normal distribution were compared using the *unpaired Student’s t-test* or the variance analysis followed by the Tukey multiple comparison, and *F test* was applied to compare the regression models. The significance level (*α*) employed in all the study was 0.05.

## Results

**Figure 2:**
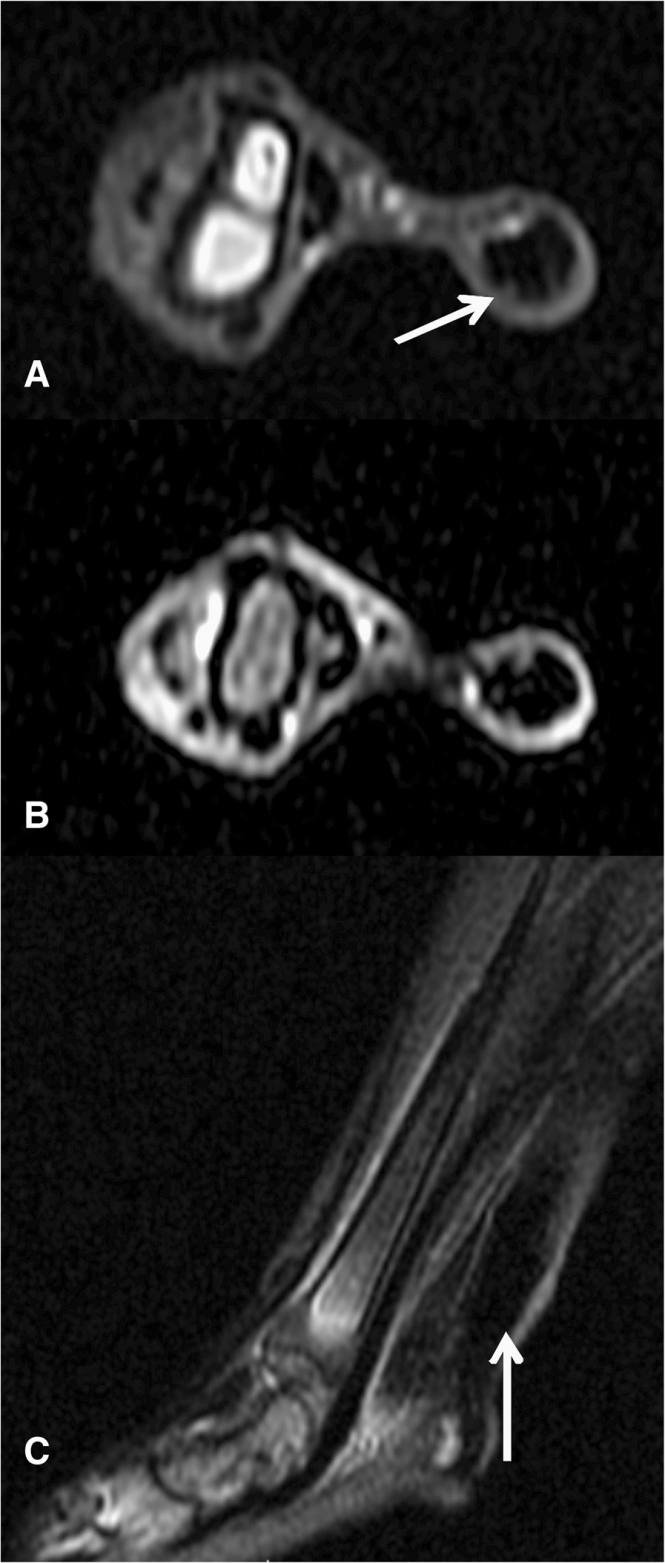
**MR images of the rabbit Achilles tendon (white arrow) from the control group 1: (A) T1 axial cut, (B) T2 axial cut with fat saturation, and (C) T2 sagittal cut with fat saturation.** Observe the homogeneity of the tendon, which is diffusely hypo-intense.

**Figure 3:**
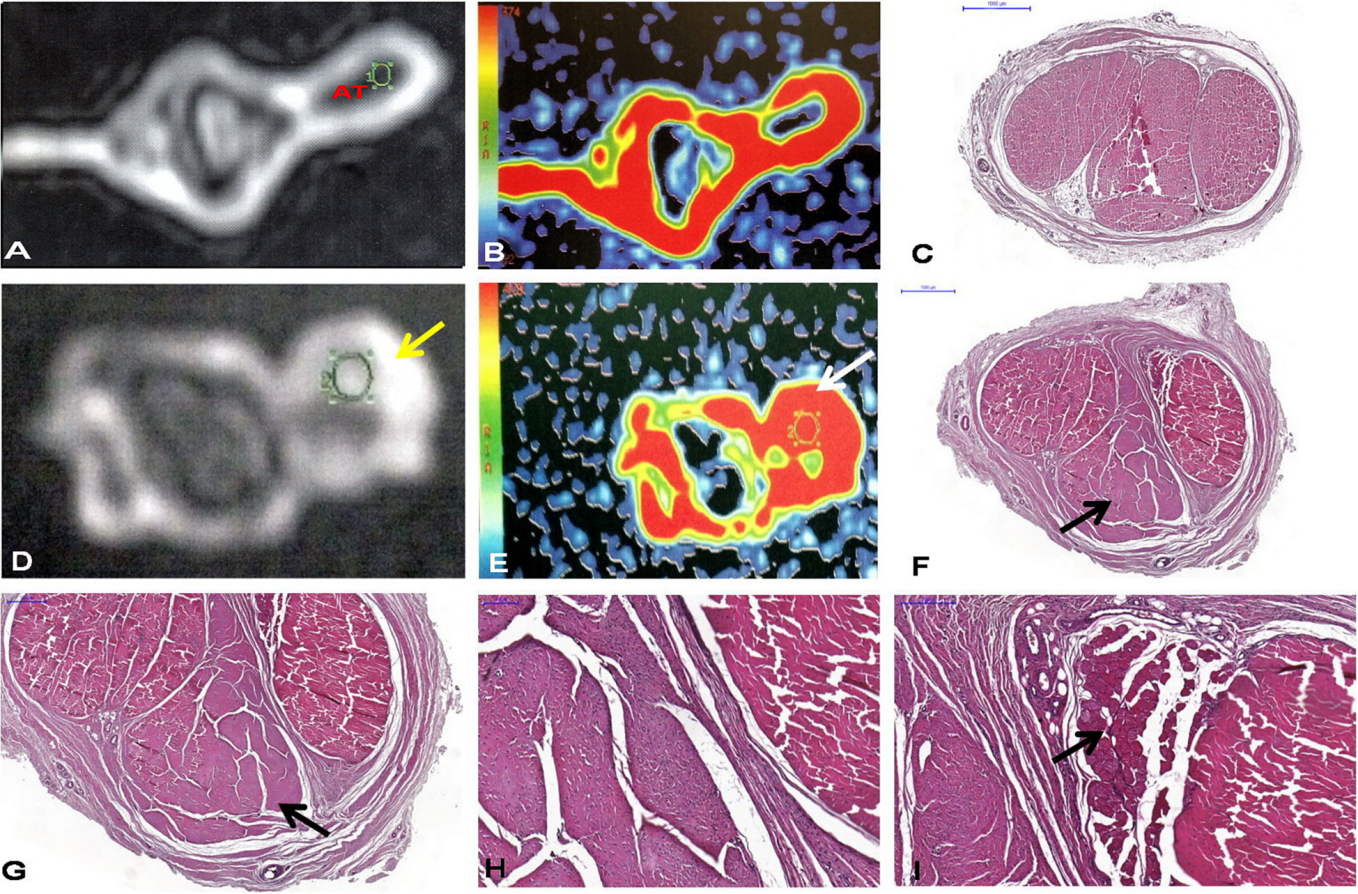
**Transversal sections of the rabbit Achilles tendon (AT) in DCE-MRI and histology.** Control group **(A-C)** and operated group **(D-F)**. Contrast enhancement in MIR in both groups corresponds to the red area on map colour with maximal contrast increase inclination. Scarring area in the operated group (yellow arrow **(D)**) corresponds to the red area on map colour (white arrow **(E)**) and pointed area on histology in the posterior region of the tendon (black arrow **(F)**). Details of the scar tissue **(G-I)**, with less organized collagen in **(G)** (arrow), hypercellularity on the left side compared to the normal tendon on the right **(H)** and preserved muscle fibres (arrow) between the scar tissue and normal tendon **(I)** (haematoxylin-eosin stain).

**Figure 4:**
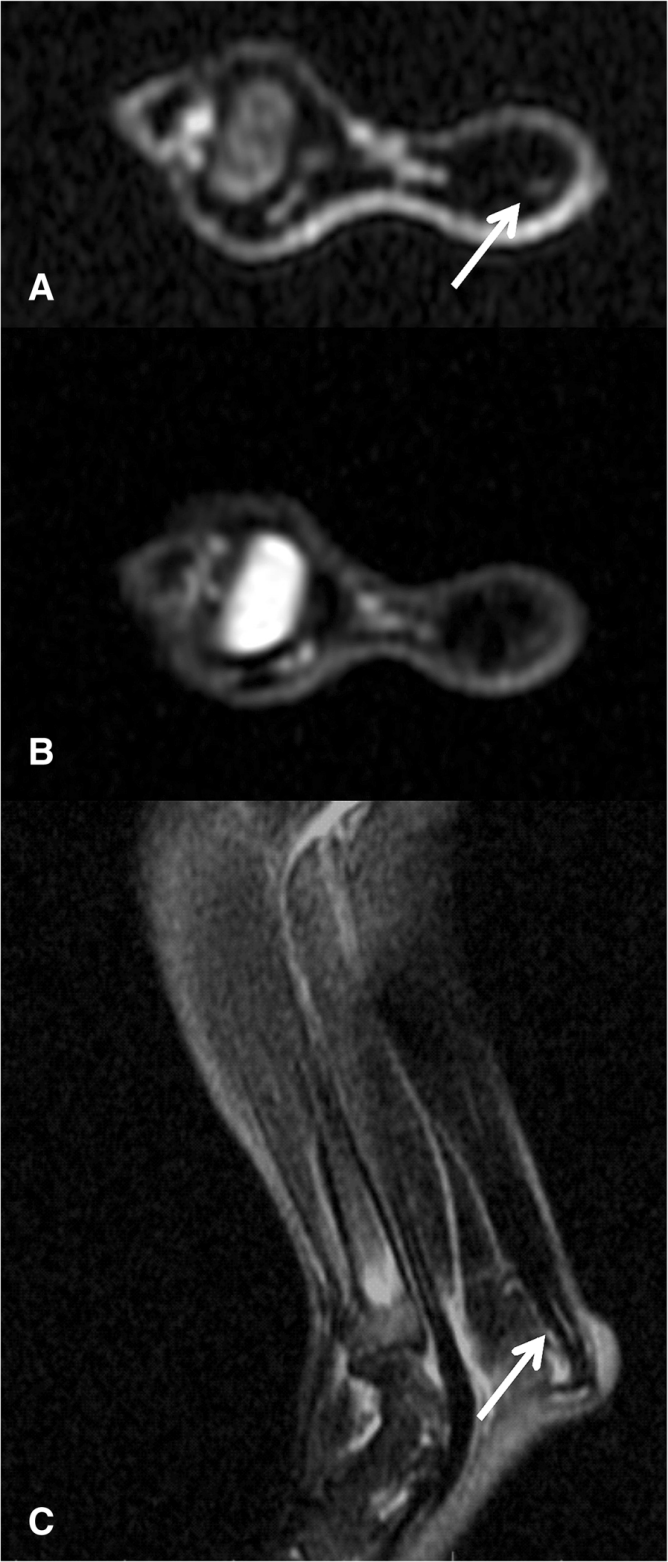
**MR images of the rabbit Achilles tendon from the surgical group 2: (A) T1 axial cut, (B) T2 axial cut with fat saturation and (C) T2 sagittal cut.** Observe the heterogeneity of the tendon, which presents hyper-intense signal focus points in T1 and T2 (arrows).

MRI in the control group revealed an Achilles tendon with homogeneous and hypo-intense aspect and regular and well-defined contours in the T1 and T2 sequences without contrast (Figure 2). There was no significant contrast enhancement in the sequence with perfusion in the control group. The tendon sheath presented a thin enhancement with the contrast (Figure 3A). The surgical group had an intense contrast enhancement in the contrast sequences and a signal intensity alteration with intra-substantial hyper-signal associated to irregular and poorly defined contours at the same sequences. There were an enlargement of the tendon in diameter and alteration in the intra-substantial signal intensity, with hyper-signal focus points and widening of the tendon sheath, which demonstrated irregular contours and intense contrast enhancement (Figures 3D and 4).

**Figure 5:**
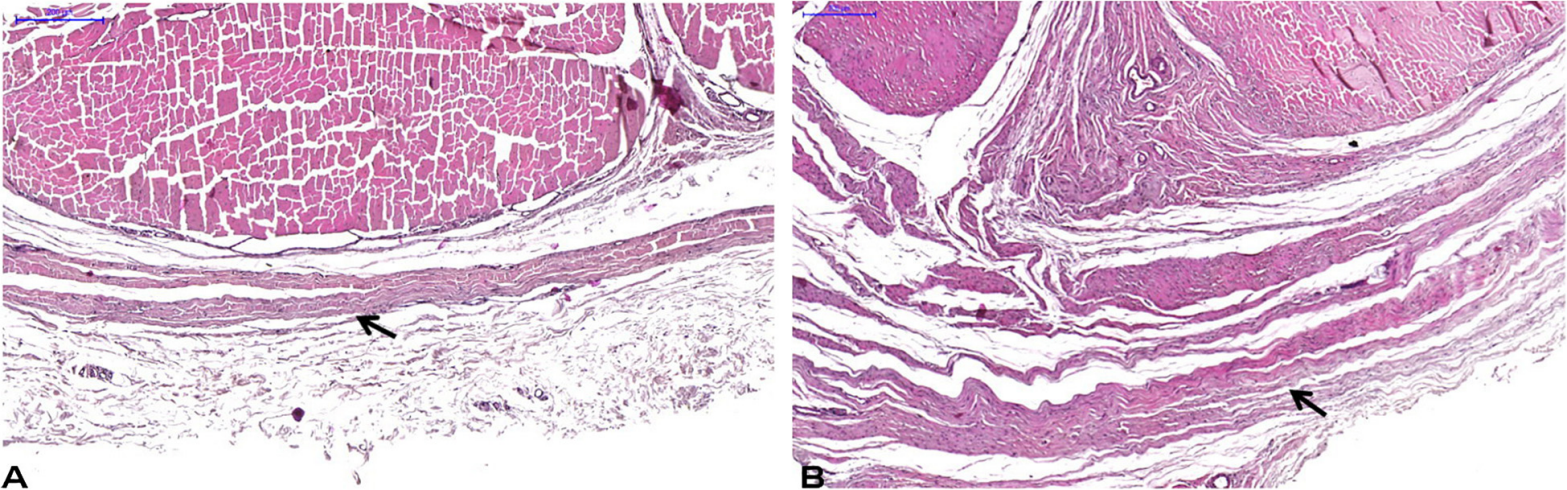
**Normal-sized Achilles tendon sheath (arrow) in the control group (A) and considerable widening of the tendon sheath (arrow) in the operated group (B) (haematoxylin-eosin stain).**

The Achilles tendon in the control group had homogeneous and hypo-intense aspect, regular and well-defined contours on MRI and organized, homogenously eosinophilic dense collagen fibres (Figure 3A,B,C). The surgical group presented a signal intensity alteration with intra-substantial hyper-signal associated to irregular wedge-shaped area corresponding to scarring tissue with disordered collagen fibres on histology (Figure 3D,E,F) and diffuse widening of the tendon sheath (Figure 5A,B).

**Figure 6:**
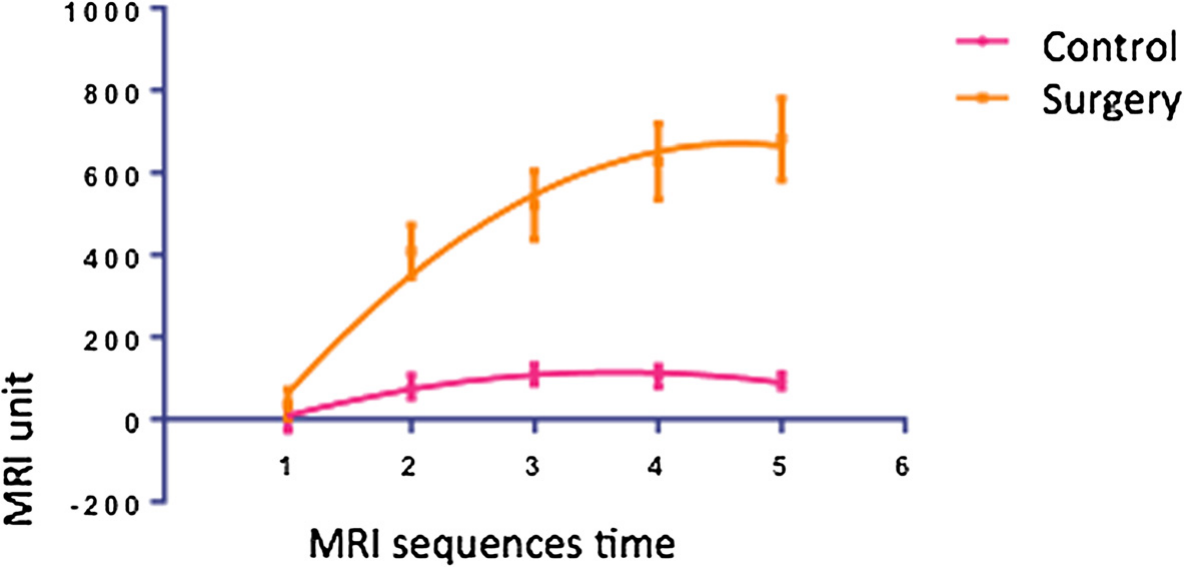
**Contrast enhancement curves in the control and surgical groups.**

There was a significant difference between the contrast enhancement curves in the control and surgical groups (*p* < 0.0001) (Figure 6). Antero-posterior and transversal diameters were bigger in the surgical group when compared to the control group (*p* < 0.05) (Table 1).

**Table 1:** **Means ± standard deviation for the antero-posterior and transversal diameter variables of the rabbit Achilles tendons**

**Parameters**	**Group 1 (control)**	**Group 2 (surgery)**
AP (mm)	4.22 ± 0.57a	5.53 ± 1.16b
T (mm)	3.32 ± 0.58a	5.53 ± 1.43b

Means with different letters (a, b) differ statistically in the Tukey post-test (*p* < 0.05).

*AP* antero-posterior diameter, *T* transversal diameter.

## Discussion

The study of the tendinopathies and of the Achilles tendon ruptures, regarding surgery and healing, depends a great deal on animal models [12,13]. Rabbit has been used to study the tendon because it is widely available, easily managed and has an adequately sized Achilles tendon, for both the histological study and MRI [14,15].

The human Achilles tendon is generally the most exposed among the inferior members [16,17]. The literature has presented results concerning the value of the MRI of Achilles tendinopathy. Karjalainen et al. [18], examining the Achilles tendon with magnetic resonance, reported its sensibility in detecting abnormalities in 94% of cases of pain in the Achilles, with an 81% specificity and 89% accuracy. An intra-observer agreement for the MRI results was good in all categories. Nonetheless, many researchers found image overlapping between symptomatic and asymptomatic individuals [19,20]. The signal of heterogeneity and subtle intra-substantial signal increases, or small focus points of T1 signal increase, may represent normal anatomy and small vessels [19]. Areas of T2 isosignal may represent tendinopathy/mucinous degeneration in asymptomatic individuals. Notwithstanding, areas of T2 hyper-signal and thickened tendons were associated to chronic symptoms [20]. Considering this, we suggest the importance of the evaluation of the image methods.

In the present study, we found a correlation between both the intra-substantial hyper-signal finding in T2 and focus points of T1 isosignal and the presence of scarring alterations in the histology. On the other hand, there was no alteration of the intra-substantial signal in the resonance neither in the histology, in the tendons of the rabbits of the control group.

Even though the rabbit has been widely used in the research of the Achilles tendon [13-15], few studies examined this tendon using MRI [21-23]. We opted to do MRI 30 days after the surgical procedure because the goal was to evaluate the scarring in the remodeling stage, which usually starts in the second and third week of the scarring process. Thus, the timing would be compatible with the minimal follow-up delay for clinical studies that use MRI to analyze its effects regarding the treatment for tendon and ligament injuries [24,25].

MRI of the Achilles tendon of the rabbit in the axial plan, with a 2-mm thickness, allows a detailed vision, which corresponds to relations observed in the sectional anatomy. Sagittal images, with a 2-mm thickness, were less useful, which may be explained by the reduced dimension of the tendons and their rotation with regard to the antero-posterior plan.

Few studies have evaluated the measure of the antero-posterior and transversal diameter as measurements of healing at the scarring area [26,27], with conflicting results. Notwithstanding, we chose to use this type of measurement because it is associated with the proportions of the image and has great interest for clinical practice.

The significant difference observed between the operated groups and the control group for the antero-posterior and transversal diameters, 30 days after the surgical intervention, suggests that the scarring process was responsible for this discrepancy. This fact was confirmed by the histological analysis, which revealed that most part of the tendon was replaced by the scarring tissue. It also showed the thickening of the tendon sheath in the surgical group.

We observed a significant statistical difference (*p* < 0.0001) between the enhancement curves resulting from the control and surgical groups. The reason for this is probably related to the fact that the normal tendon (control group) does not usually present significant contrast enhancement, since it is less vascularized, even in histological studies, which show that its vascularization is provided mainly by the tendon sheath [28].

The main finding of this study was the difference between the surgical and control groups regarding the contrast enhancement peak in the dynamic DCE-MRI of the Achilles tendon. The mechanism used to observe the contrast enhancement in tendinopathies and during the post-operative period is not absolutely understood. Contrast agent firstly distributes itself inside the vascular compartment and then spreads to the extracellular interstitial. The increase of vascularization and/or vascular permeability, or even the increase of the volume of the interstitial stroma, may explain the greater enhancement in the pathological areas and not in the surrounding normal tissue, as was demonstrated in the comparison between the resonance images and the histological analysis [29,30].

Increased extracellular matrix is a frequent finding in tendinopathies, such as those that affect the Achilles tendon, the rotator cuff, the origin of the extensor carpi radialis brevis (lateral epicondylitis) and the patellar tendon (*jumpers knee*) [31,32]. It reveals, furthermore, the disorganization of the collagenous fibres and a vascularization and cellularity increase, but do not show the presence of any inflammatory infiltrate cells [33]. These results are similar to the ones found in our study with animals, which seems to corroborate the potential of the investigation with DCE-MRI in patients with tendinopathies.

There are possible limitations to the study design that we would like to address. In our analysis, diameter measurements were used only as quantitative variables for the evaluation of the tendon healing. Differences in the expression of other potential markers, such as fibrosis and angiogenesis, could also translate qualitative result of healing. Functional studies may also be useful for better interpretation of histological and image results. However, these measurements were not performed at this stage of our experiment.

## Conclusion

DCE-MRI is a technique that has the potential of demonstrating alterations in the operated Achilles tendon, revealing, in relation to the control group, different patterns of contrast enhancement.
